# Intergenerational Communication as Non-Pharmacological Care in a Japanese Nursing Home

**DOI:** 10.1093/geroni/igaa057.1572

**Published:** 2020-12-16

**Authors:** Toshiko Hamaguchi

**Affiliations:** The University of the Sacred Heart, Tokyo, Japan

## Abstract

Statistics shows that Japan now has 28.4% of population aged over 65, which marks the highest in the world. Moreover, one in four over 65 is said to have ADRD or MCI. Traditional family caregiving derived from filial piety is giving way to moving to care facilities in order to reduce a burden on children or get professional care. This suggests that everyday communication for older adults involves younger conversational partners with varying degrees of shared knowledge and experiences. Such intergenerational communication can be challenging and stressful on both sides. To date, empirical studies that observe interactional strategies of both younger caregivers and older adults is unknown. Using 21 recordings of weekly conversational activities (45-60 minutes) led by recreation workers (female, 40s) taken at a nursing home in Japan, this qualitative study demonstrates how (1) sharing of the past and present becomes a learning opportunity for both recreation workers and older participants with ADRD (aged 87-95) , (2) reference to nursing-home living establishes and reaffirms their interpersonal relationship, and (3) intergenerational conversation becomes a tool to express life satisfaction for the older participants. This evidence-based study proposes mundane conversations during recreational activities as part of non-pharmacological person-centered care which serves to improve the quality of life and life satisfaction of older adults at care facilities. It is therefore important to study communicative strategies of professional caregivers since they affect social engagement as well as emotional and psychological well-being of those at the end-of-life stage.

